# Children do not distinguish efficient from inefficient actions during observation

**DOI:** 10.1038/s41598-021-97354-9

**Published:** 2021-09-13

**Authors:** Ori Ossmy, Danyang Han, Brianna E. Kaplan, Melody Xu, Catherine Bianco, Roy Mukamel, Karen E. Adolph

**Affiliations:** 1 Department of Psychology, Center for Neural Science, New York University, 6 Washington Place, Room 403, New York, NY 10003 USA; 2 School of Psychological Sciences, Sagol School of Neuroscience, Tel-Aviv University, Tel Aviv, Israel

**Keywords:** Human behaviour, Perception

## Abstract

Observation is a powerful way to learn efficient actions from others. However, the role of observers’ motor skill in assessing efficiency of others is unknown. Preschoolers are notoriously poor at performing multi-step actions like grasping the handle of a tool. Preschoolers (*N* = 22) and adults (*N* = 22) watched video-recorded actors perform efficient and inefficient tool use. Eye tracking showed that preschoolers and adults looked equally long at the videos, but adults looked longer than children at how actors grasped the tool. Deep learning analyses of participants’ eye gaze distinguished efficient from inefficient grasps for adults, but not for children. Moreover, only adults showed differential action-related pupil dilation and neural activity (suppressed oscillation power in the mu frequency) while observing efficient vs. inefficient grasps. Thus, children observe multi-step actions without “seeing” whether the initial step is efficient. Findings suggest that observer’s own motor efficiency determines whether they can perceive action efficiency in others.

## Introduction

Observing other people’s behavior has a powerful effect on observers’ own behavior. Mere action observation—perceiving without doing—modifies parameters such as movement trajectory and velocity^1–3^ and grip and squeeze forces^4,5^. Indeed, people’s own actions can become more efficient after observing someone else performing the same actions^6,7^.

Nonetheless, observing and doing actions provide very different sources of information about action efficiency. When performing an action, people physically *experience* the efficiency of the action through perceptual feedback. Thus, people can assess the efficiency of their actions and facilitate motor control by comparing alternative actions and selecting the most efficient option for the specific situation^8^. However, when observing other people’s actions, observers do not physically experience whether the action is efficient or not. Instead, they must estimate efficiency based solely on visual information^9^. Motor-cognition theories^10^ propose that seeing an action activates the neural motor program used when performing the action^6,11^. So, observers can facilitate their own motor control by comparing the observed action to the neural representation in their motor system^12–14^.

Despite the importance of observers’ own motor skills for learning by doing, the question is still open regarding the importance of observers’ motor skills for learning via observation. That is, can observers, who are not efficient actors themselves, distinguish efficient from inefficient actions in others? The answer is important for identifying the features of observed actions that are encoded in observers’ neural motor system and for understanding how action observation facilitates motor learning. Presumably, to obtain the necessary information about efficiency, observers must direct their gaze to the relevant action at the appropriate time. And to differentiate efficient from inefficient actions, observers’ brains must register the visual information they obtain.

**Figure 1:**
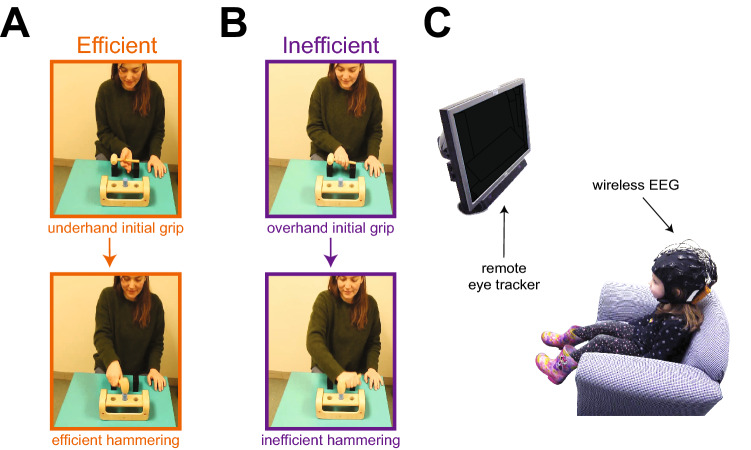
Displays and experimental set up. Participants observed an adult actor pound a peg using a hammer and a pegboard. Video displays were designed to distinguish perception of efficient versus inefficient means of using the hammer to pound the peg. In all displays, the action started with the hammer handle pointing away from the dominant hand. (**A**) In the “efficient” displays, the action began with an underhand, radial grip that led to a comfortable overhand, radial grip to pound the peg. This is the grip used by adults in previous work. (**B**) In the “inefficient” displays, the action began with a habitual, overhand, ulnar grip that led to inefficient tool use. This is the grip used by children in previous work. (**C**) Experimental set up for children with remote eye tracker and wireless EEG. Adults sat on an adult-sized chair.

Here, we addressed the question of whether inefficient actors can distinguish efficient from inefficient actions in others by exploiting children’s inefficient performance in multi-step actions like tool use. Previous work showed that children fare poorly when efficient tool use requires the initial movement to take the future end goal into account^15^. For example, to pound a peg when the handle of a hammer points away from their dominant hand (shown in Fig. 1A), adults use an atypical, underhand grip to grasp the hammer to enable a smooth transition to efficient hammering^16,17^. In contrast, children repeatedly use a habitual overhand grip, so they end up holding the handle in an inefficient awkward grasp position to pound the peg (Fig. 1B) and must subsequently adjust their grip^16,17^. Here, we tested children at similar ages (3–5 years) when they typically use an inefficient initial grip to grasp the handle of a hammer. Children and adults observed videos of actors using efficient and inefficient initial grips to grasp a hammer to pound a peg (compare Fig. 1A, B and videos at databrary.org/volume/321/slot/44641/-?asset=231554 and databrary.org/volume/321/slot/44643/-?asset=231573).

We tested three potential outcomes: (1) If efficient action performance is unnecessary to distinguish efficient from inefficient actions in others, then both adults and children should obtain the relevant visual information by directing their gaze to the actor’s initial grip and both groups should show differential physiological responses to the information they obtain. (2) However, if efficient action performance is required to distinguish efficient from inefficient actions in others, children should fail to direct their gaze to the actor’s initial grip and/or fail to differentiate the grips in their physiological responses. Note that this outcome supports the “motor-resonance” theory—that observers’ own motor experiences contribute to perception of others’ actions^6,10^—only if children both look at the actions and show physiological responses to the actions; that is, children must provide evidence that they attended to the videos. (3) Of course, children differ from adults in many important ways beyond the efficiency of their manual actions (visual attention to a task, information-processing time, etc.). Thus, if children do not attend to the videos or do not show physiological responses to the displays, we must conclude that the experiment failed to adequately address the question of mandatory links between action performance and action observation for correctly evaluating action efficiency in others.

We used eye tracking to assess visual attention to each part of the action—where each participant looked and when. In particular, we asked whether both children and adults looked at the relevant part of the scene at the appropriate time to gather visual information about the efficiency of actors’ initial grip before pounding the peg (i.e., looking at the hammer and hand during actors’ reach and grasp). We used deep artificial neural networks and probability maps to test whether participants’ looking patterns distinguished efficient from inefficient grasps. We used pupil dilation and electroencephalography (EEG) to test whether participants’ physiological responses provided evidence of registering differences between efficient and inefficient grips^18^, that is, whether participants “see” as well as look at the initial step in a multi-step action. Accumulated time looking at the displays and physiological responses to the displays provide evidence of overall visual attention.

## Results

Overall, children and adults looked equally long at the displays during the reach and initial grasp and during the pounding of the peg (*p*s > 0.10 for all looking measures). Likewise, children and adults looked equally long at displays of the efficient and inefficient actions (all *p*s > 0.10). However, the groups were only similar when we analyzed accumulated looking time. As we report below, the two groups differed in real-time patterns of looking—where participants looked and when as the event unfolded–and in their physiological responses to efficient and inefficient actions.

### Looking at areas of interest relevant for assessing action efficiency

**Figure 2:**
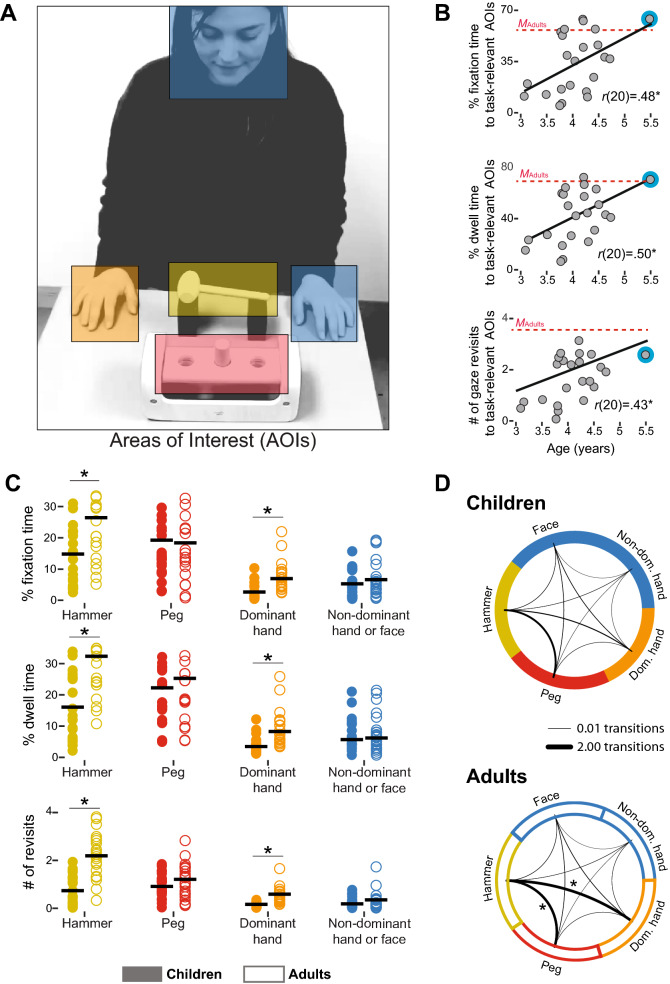
Looking at areas of interest (AOIs). (**A**) Areas of interest for eye-tracking analyses. We calculated amount of looking to 5 AOIs during each video: 3 task-relevant AOIs—hammer (yellow), peg (red), dominant hand (orange), and 2 task-irrelevant AOIs —the face and the non-dominant hand (blue). Right-handed participants observed displays of actors using their right hand to pound the peg, and vice versa for left-handed participants. (**B**) Correlations between children’s age and measures of looking to the AOIs: percent fixation time, percent dwell time, and number of revisits (*p*s < .05). Red dotted lines indicate adult averages. After excluding the outlier (marked in blue), correlations remained significant, *r*s(19) > .43, *p*s < .05. (**C**) Measures of looking to AOIs for each group averaged across videos and trials. For fixation time, dwell time, and number of revisits, adults looked significantly more at the hammer and the dominant hand (all *p*s < .01) compared to children. (**D**) Transitions between each pair of AOIs. Line thickness denotes the average number of transitions between AOIs. Compared to children, adults shifted their gaze more between the dominant hand and the hammer (*p* < .01), and between the hammer and the peg (*p* < .05).

Despite looking at the displays, for most children, looking at the relevant areas of interest (AOIs shown by warm colors in Fig. 2A) was lower than adult levels (red lines in Fig. 2B). However, every measure of looking—fixation time, dwell time, and gaze revisits—increased with age; *r*s(20) > 0.43, *p*s < 0.05; scatters in Fig. 2B. Correlations with age remained significant after removing the oldest child, *r*s(19) > 0.43, *p*s < 0.05.

As shown in Fig. 2C, children and adults looked equally long at the final goal target (peg), but children looked less at the areas relevant for action efficiency (hammer and dominant hand). ANOVAs on fixation time, percent dwell time, and number of revisits, using 2 (ages) × 2 (efficient vs. inefficient actions) × 5 (AOIs) as factors, showed main effects only for age and AOI, and interactions between age and AOI (Table 1). Sidak-corrected post hoc comparisons confirmed higher percentages for all measures of looking at the hammer and dominant hand in adults than children, and no age differences in looking at the peg and non-relevant AOIs (face and non-dominant hand).

**Table 1:** Gaze-shifts.

	Fixation time		Dwell time		Revisits	
Age	*F*(1,440) = 26.31	*p* < .00*	*F*(1,440) = 21.63	*p* < .00*	*F*(1,440) = 74.57	*p* < .00*
Action	*F*(1,440) = .61	*p* = .43	*F*(1,440) = .20	*p* = .65	*F*(1,440) = .72	*p* = .39
AOI	*F*(3,440) = 103.81	*p* < .00*	*F*(3,440) = 86.92	*p* < .00*	*F*(3,440) = 136.95	*p* < .00*
Age × action	*F*(1,440) = .00	*p* = .98	*F*(1,440) = .00	*p* = .97	*F*(1,440) = .00	*p* = .99
Age × AOI	*F*(3,440) = 9.70	*p* < .00*	*F*(3,440) = 8.24	*p* < .00*	*F*(3,440) = 30.77	*p* < .00*
AOI × action	*F*(3,440) = .12	*p* = .94	*F*(3,440) = .10	*p* = .98	*F*(3,440) = .44	*p* = .72
Age × action × AOI	*F*(3,440) = .52	*p* = .66	*F*(3,440) = .39	*p* = .81	*F*(3,440) = .13	*p* = .94

2 (ages) × 10 (pairs of AOIs) ANOVA results for gaze shifts between AOIs.

Adults also showed more gaze shifts among relevant AOIs than children. Each vertex in Fig. 2D represents an AOI, and the thickness of each black line represents the average number of transitions between the pair of AOIs in both directions (e.g., a gaze shift from the dominant hand to the hammer and a gaze shift from the hammer to the dominant hand count as 2 transitions between these AOIs). A 2 (ages) × 10 (pairs of AOIs) ANOVA confirmed main effects for age, pair, and an interaction between age and pair (Table 2). Sidak-corrected post hoc tests showed that the hammer-to-peg and hammer-to-dominant-hand transitions were higher in adults compared to children.

**Table 2:** AOI analysis.

Age	*F*(1,440) = 19.68	*p* < .00*
AOI pairs	*F*(9,440) = 133.37	*p* < .00*
Age × AOI pairs	*F*(9,440) = 5.62	*p* < .00*

2 (ages) × 2 (actions) × 5 (AOIs) ANOVA results for looking measures (fixation time, percent dwell time, and number of revisits). Asterisks denote significant differences.

### Looking at the right thing at the right time

**Figure 3:**
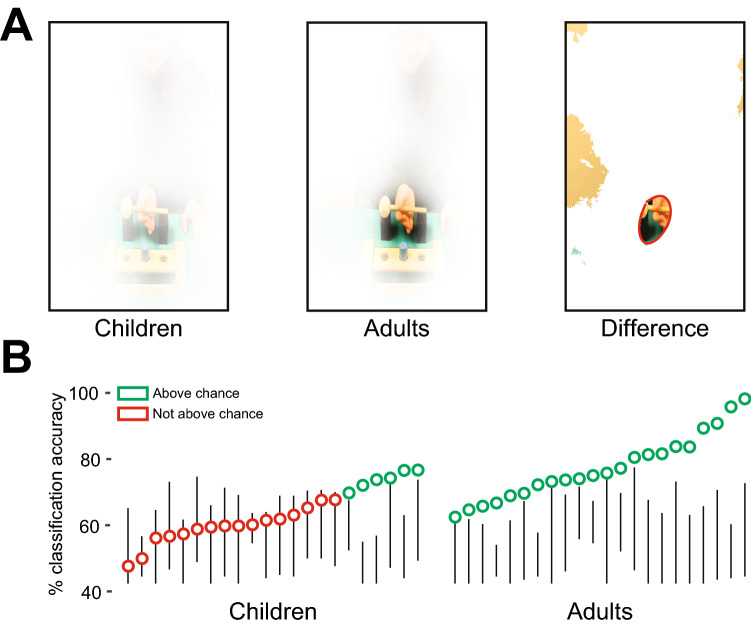
Looking patterns. (**A**) Probability maps representing the likelihood that children (left) and adults (middle) fixated each pixel during reaching (time interval from movement onset to the initial grasp). If the pixel is white, then the group’s probability of fixating that pixel is 0. As the probability increased, the pixel is clearer. When the probability is 1, the pixel is shown in its entirety. The right map represents the difference between the two probability maps. If the pixel is white, there is no significant difference between the two groups. If the video frame is shown in the pixel, then adults fixated more on this pixel than children did. The red circle denotes significant differences after correction for multiple comparisons. (**B**) Deep-learning classification of efficient and inefficient "looking videos" of individual participants. In the adult group, classification was significant for all 22 participants; whereas in the child group, classification was significant only for 6 participants. Participants are ordered by accuracy. Black lines indicate range of shuffle accuracy for each participant. Each symbol represents accuracy based on the real labels. Symbols located along the line show insignificant accuracy relative to chance.

Accumulated measures of looking do not necessarily reflect the temporal dynamics of looking in the key moments of the action. To obtain visual information about action efficiency, observers must look at the relevant AOIs at the right time in the action sequence. Thus, we performed two real-time analyses—temporal probability maps and deep learning (see Methods). Figure 3A visually illustrates the average temporal probability map across all video frames (from movement onset to initial grip) for each age group by varying the opacity of each pixel. If the average probability of fixating a specific pixel is 0 (i.e., no participant fixated that pixel), the pixel is completely opaque (as in the outer regions of the child and adult maps). The pixel becomes less opaque as the probability of fixation increases (as in the hammer region of the adult map). When the probability of fixating a specific pixel is 1 (i.e., every participant fixated that pixel), the pixel is completely transparent, and the display is clearly visible in the figure. Group comparisons for each pixel showed significant differences inside the circular region in the difference map (map at far right of Fig. 3A); *t*s(42) > 3.58, *p*s < 0.05, false discovery rate corrected for multiple comparisons). In other words, adults looked more than children at the pixels inside the red circle when the actor reached out and grasped the handle, meaning that adults were more likely than children to fixate the handle of the hammer when the actor initially reached and grasped it.

Deep-learning analysis successfully distinguished efficient from inefficient planning based on the "looking videos" (see Methods) of every adult—that is, where adults looked as the event unfolded. In contrast, the analysis successfully distinguished looking videos only for a small subset of children (6 of 22). As shown in Fig. 3B, the mean accuracy across participants of the integrated CNN (convolutional neural network) and LSTM (long-short term memory) classifier for the type of action (efficient vs. inefficient) was *M* = 76.2%, *SD* = 11.59, when it was trained on adults’ looking patterns, but only *M* = 64.9%, *SD* = 8.32, when it was trained on children’s looking patterns. Classification accuracy in the children was unrelated to their age *r*(20) = 0.21, *p* = 0.34. These findings show that children and adults gathered different visual information as expressed by differences in the temporal dynamics of their looking patterns.

### Pupil dilation

**Figure 4:**
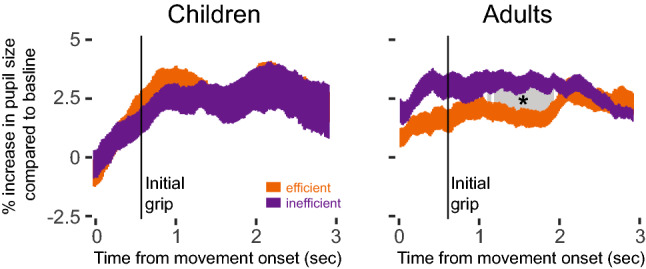
Pupillary responses to each display after movement onset in children (left panel) and adults (right panel). The change in children’s pupils was not significantly different during observation of videos of efficient versus inefficient actions. Adults’ pupil size was larger when observing inefficient planning compared to efficient planning starting about 1.1 seconds after the initial grip. Gray area denotes significant differences (false discovery rate corrected for multiple comparisons).

Pupil dilation reflects observers’ physiological responses to the displays. Adults showed greater pupil dilation when observing inefficient planning than when observing efficient planning at *M* = 0.7–1.4 s after the moment of initial grip (Fig. 4, gray area); *t*s(42) > 2.02, *p*s < 0.05. Increased pupil dilation was absent in children (Fig. 4; *t*s(42) < 0.83, *p*s > 0.40). Given that the known delay in pupil dilation is 1–1.5 s after the visual event^18,19^, this finding indicates a difference in adults’ attention to the different grips. The lack of difference in children’s pupillary response in the moments following the initial grasp corroborates failure of deep learning analyses to classify efficiency based on children’s looking videos.

### Neural activity

We analyzed the neural data in two stages—localization and classification. In the localization stage, we used the data obtained during observation of two additional videos to identify frequencies, times, and electrodes in which neural activity was sensitive to action observation. A 2 (ages) × 2 (test video or localizer video) ANOVA for looking measures (fixation time, percent dwell time, and number of revisits) on task-relevant areas validated that participants looked at the localizer videos similarly to the test videos. We found no main effect of video type *F*s(1,175) = 0.72, *p*s > 0.39, and no interaction between age and video type *F*s(1,175) = 0.81, *p*s > 0.36.

**Figure 5:**
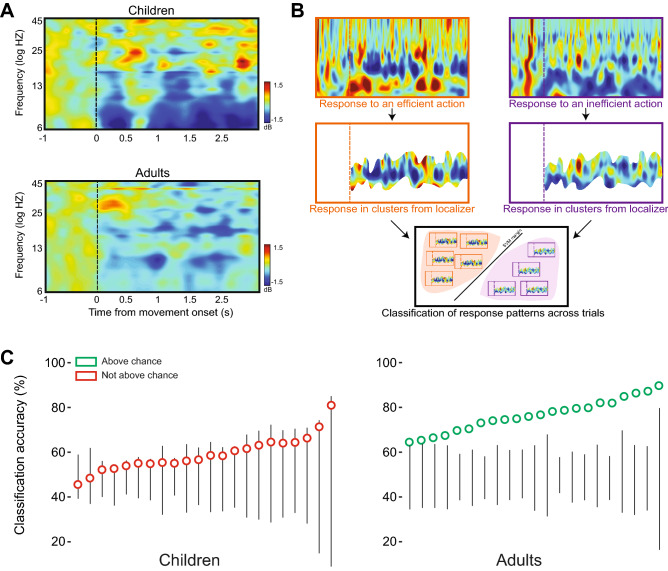
EEG results. (**A**) Event-related spectral perturbation map representing average changes in oscillation power across sensorimotor electrodes of children (top) and adults (bottom), collapsed across localizer videos. (**B**) Classification of action-related neural responses. For each participant, we used a SVM classifier to differentiate neural responses evoked by the two types of displays. We used the channels, frequencies and times of suppression that were identified in the localizer stage. (**C**) Classification analysis between efficient and inefficient action videos revealed that in the adult group, every participant showed significantly greater than chance differences in neural activity (right panel). In contrast, in the child group, no child showed classification levels significantly different from chance (left panel). Participants are ordered by accuracy. Black lines indicate range of shuffle accuracy for each participant. Each symbol represents accuracy based on the real labels. Symbols located along the line show insignificant accuracy relative to chance.

We used a data-driven approach using nonparametric cluster analysis to examine significant event-related spectral perturbation (ERSP) locked to movement onset in all participants and electrodes. Table 3 shows the frequencies of the individual EEG signal in which oscillation power in sensorimotor sites was significantly suppressed relative to baseline. The average multi-participant ERSP maps for each group (Fig. 5A) are consistent with previous studies^20^ and confirm activity suppression in the mu band (children: 6–9 Hz, *M* = −0.94 *SD* = 0.86, *t*(21) = 5.13, *p* < 0.00; adults: 8–13 Hz, *M* = −0.42 *SD* = 0.6, *t*(21) = 3.23, *p* < 0.00; unequal variance *t*-test compared to zero). These findings indicate increased event-related desynchronization of the EEG signal in both groups, following observation of others’ actions.

**Table 3:** Localizer results.

Children			Adults		
*# channels*	*# clusters*	*Frequency range*	*# channels*	*# clusters*	*Frequency range*
1	2	6.40–8.19	3	4.66	5.85–25.00
1	3	6.03–13.75	4	1.75	5.85–12.78
4	2	5.85–13.70	1	2	8.80–17.23
1	7	6.68–19.51	4	2.50	7.03–22.24
1	3	6.85–19.65	5	5.40	6.03–23.07
1	1	5.85–7.03	10	3.90	6.03–25.00
2	8.50	5.85–18.95	4	2.75	6.07–25.00
7	7.71	5.85–25.00	2	7.50	6.53–24.70
2	7	5.85–24.81	1	1	11.45–13.16
1	1	6.44–7.61	1	2	12.32–25.00
1	1	6.97–7.78	1	3	6.85–18.13
1	1	7.78–9.34	2	5	6.85–21.60
2	7.50	5.85–25.00	1	3	5.85–25.00
1	18	5.85–25.00	2	4	5.98–15.90
1	5	7.50–21.29	5	3.20	5.85–24.60
1	4	6.03–11.79	1	11	6.25–25.00
3	2.33	5.85–22.08	8	3.12	6.85–12.97
2	2.50	6.03–13.06	7	4.85	5.85–20.53
1	4	7.85–20.68	1	7	13.16–23.07
1	6	5.85–21.92	4	8.50	6.85–25.00
2	4	6.63–18.54	1	5	6.85–25.00
1	4	6.44–21.14	1	4	12.41–23.58

Cluster details for each participant in each group. *# channels* denotes the number of motor channels with at least one significant cluster. *# of clusters* denotes the average number of clusters per channel. *Frequency range* is the minimal and maximal frequencies for each participant’s clusters.

As with pupil dilation, classification analyses showed differential EEG responses in adults to efficient versus inefficient initial grips, but not in children. In the classification stage (Fig. 5B), we examined whether neural signals evoked by the test videos in the areas defined by the individual subject localizer distinguish between efficient and inefficient actions. Adults showed significant above-chance classification performance (*M* = 79.2%; Fig. 5C). However, children did not show significant neural differentiation between the two actions (*M* = 57.3%).

## Discussion

A novel combination of methods (eye tracking, pupillary responses, EEG, and machine learning) confirmed that observers’ motor efficiency affects their perception of efficient and inefficient actions. Specifically, at an age when children have difficulty planning efficient multi-step actions, they also have difficulty differentiating the efficiency of other people’s actions while grasping a hammer to pound a peg. On every measure, adults showed clear evidence of differentiating action efficiency, but preschoolers did not.

### Motor skills facilitate action perception

Previous developmental research focused on how infants look at action goals^21–23^ and whether they see when one goal differs from another. For example, researchers found that infants looked longer at an actor pulling a cloth to retrieve a toy after seeing the same action with a different toy^24–26^. Infants also show increased pupil dilation when viewing an unexpected change in a goal (moving food-laden spoon to the hand rather than mouth^27^) and increased EEG activity in the gamma frequency band over primary motor cortex^28^ for incomplete actions (end goal not achieved) compared to completed actions (end goal achieved).

In contrast to previous work, the current study focused on how young children look at the action *means*. Previous developmental work on the perception of action means focused primarily on infants and yields discrepant findings. In some studies, infants showed evidence of perceiving the means to achieve a goal. For example, 9-month-olds looked longer when grip aperture of a reaching hand (the means) and the size of a cup (goal target) were unmatched versus matched^29^. Likewise, eye-tracking showed that 6- to 20-month-olds anticipate the goal based solely on how actors wield a tool^30,31^. Moreover, 12- and 14-month-olds imitated unusual means to achieve a goal (turning on a light with their head rather than their hand), but only when the action was more efficient because the actor’s hands were occupied^32,33^. Yet, other studies found that infants and children do not attend to the efficiency of means to achieve a goal. Ten- to 12-month-olds showed no difference in looking time when actors changed the means to achieve a goal by pulling a different cloth or lifting a different box^34,35^. Similarly, 12- and 18-month-olds imitated the end goal of putting a mouse in a house^36^, but not the means to get there (hopping motions and distinctive sounds). Three- to 5-year-olds^37^ imitated only the observed action goal (touching hand to ear) and not the means to achieve it (which hand touched target ear).

Our displays were designed to examine the effect of motor skills on perceiving the efficiency of action *means*. In prior work on action production^16^, adults used the efficient means (grasping action) nearly uniformly: They used initial overhand grips when the hammer handle pointed toward their dominant hand and underhand grips when the handle pointed toward their non-dominant hand^38^. In contrast, preschoolers rarely performed the efficient, underhand grip when the hammer handle pointed toward their non-dominant hand. Instead, they used the same overhand grip on most trials, so they primarily executed the inefficient action^16,17^. In the current study, only adults showed differential looking patterns and neural activity between efficient and inefficient actions. These findings, as predicted by the motor resonance theory of action perception^10^, suggest that the activation of observers’ own motor programs is built from their motor experiences^39,40^, and so motor resonance generated differential responses only in adults.

Finally, our findings have implications for understanding how children learn from observation because they raise an important question: If children, as inefficient actors, cannot distinguish efficient from inefficient actions (when the goal is achieved in both), can they learn efficient actions by mere observation? Future research should address this question by training children in observing actions that are not in their repertoire and testing their efficiency. Future work should also investigate other factors in the display that might affect learning from observation such as the actors’ age and gender and the type of action performed (manual, locomotion, with or without objects).

### Looking without seeing

Use of eye tracking, pupillary responses, and EEG allowed us to obtain evidence about where participants directed their gaze and what they “saw” or differentiated. Children looked at the end goal (peg) just as much as adults did. However, adults gathered *more* relevant visual information about the initial step in this multi-step action sequence as evidenced by longer looking to the performing hand and hammer, more gaze shifts between them, and more fixations on key areas that distinguished efficient from inefficient means.

Children, like adults, showed physiological responses to the displays, demonstrating that they indeed looked at the displays and paid attention to the actions. They showed increased pupil dilation (Fig. 4) and stronger mu suppression (Fig. 5A) after movement onset. However, unlike adults who exhibited differences in pupillary response and action-related neural activity when they observed efficient versus inefficient grasping actions, children showed the same response, regardless of condition. These findings suggest two related explanations for children’s failure to distinguish efficient from inefficient actions. First, children do not look at the right thing at the right time, so they cannot distinguish differences in the actors’ initial grip. Second, even if children had gathered the relevant visual information in the moments surrounding the grasp (and a handful of children did), their brains failed to process it adequately to notice differences in efficiency. In other words, looking is necessary but not sufficient to ensure that the relevant information is processed.

Indeed, our findings show that children’s action-related neural activity while observing actions does not guarantee that they registered the way the action was performed. First, children and adults differed in their pupillary dilation. Changes in pupil size reflect changes in the activity of neuromodulatory brainstem centers that relate to several cognitive functions and various mental states (e.g., “surprise” after violation of expectation.)^18,41,42^ Because we tested participants’ reactions to the displays using multiple measures (looking time, looking patterns, EEG), and participants’ responses were not related to mental states, it is more likely that group differences in pupillary responses arise from differences in the cognitive mechanisms underlying action observation.

Moreover, we found suppressed oscillation power in the mu frequency of the EEG signal in response to action observation. This finding supports previous evidence showing that observed actions are simulated in the observers’ own motor system^20,43^. However, unlike adults, children’s pattern of activity did not differ *between* efficient and inefficient means. This finding points to an important, unanswered question regarding action-related neural activity: What features of the observed action are encoded in children’s mu suppression and how do these features change with development? Addressing this question in future research will improve understanding of mechanisms underlying children’s imitation and what they learn when they observe others.

Finally, the current study sheds light on how children perceive efficient actions but does not directly address the developmental question of whether perceiving efficiency precedes the ability to produce efficient actions. Some studies suggest that performing a motor action precedes or facilitates action perception^44,45^ whereas other studies suggest that perception precedes action production^46^ or that both develop simultaneously^47^. Unfortunately, with the current study design, testing how children execute a task before perceiving the same task would have created a perception bias, and testing the children after perception would have created an execution bias. We tested children at ages when they are inefficient in the hammering task^16^, but further research is needed to test whether children who are efficient in producing actions react differently to observing efficient versus inefficient actions.

### The cost of anticipatory looking

We offer an additional suggestion. Children’s failure to notice differences in actors’ initial grip might result from their prediction of the end goal. Similar to previous eye-tracking studies^27,48,49^, we found that children shift their gaze to the actor’s goal target (here, the peg) *before* the action is completed. Possibly, early gaze shifts come with a cost. If young children look at the goal target too soon—without the frequent gaze shifts between peg and hammer displayed by adults—children may fail to notice the initial grip. Thus, anticipatory eye movements reveal children’s ability to understand others’ motivations and intentions but may also indicate a failure to perceive how others performed the action.

## Conclusion

We provide evidence that children fail to register elements in how others perform multi-step actions. They do not distinguish efficient from inefficient actions. Children also show deficits in planning and executing such actions themselves. Our findings suggest that observers’ own motor experience underlies the perception of action efficiency.

## Methods

Videos of the displays, de-identified looking videos of the eye-tracking recordings, and EEG data are publicly available in the Databrary web-based library (databrary.org/volume/321). With participants’ permission, third-person videos of their behaviors, individualized eye-tracking and EEG, and demographic data are also shared in Databrary with authorized researchers. The analysis codes are shared on Github (https://anonymous.4open.science/r/321/).

### Participants

We tested 22 children from 3.09 to 5.49 years of age (*M* = 4.06 years; 11 girls) and 22 adults from 19.37 to 26.40 years of age (*M* = 22.02 years; 14 women). Children were recruited from a pool of families in the NYC area who had expressed interest in participating in research. Adults were recruited through word of mouth. Most participants were white and middle class. Children received a robot toy, photograph magnet, and tote bag as souvenirs of participation, and adults received a photo magnet. Data from 10 additional children were not analyzed because they did not attend to the displays (6 children) or refused to wear the EEG cap (4 children). Data from an additional 7 adults and 10 children were excluded due to experimenter error in syncing the EEG with eye tracking and displays (see participants labeled “excluded” in the shared Databrary volume). The experiment conformed to the guidelines approved by the ethics committee at New York University.

All participants were healthy with normal vision. We verified hand dominance so as to match the actor’s dominant hand in the video displays to each participant’s dominant hand. All adults reported one dominant hand (right = 20, left = 2). All children also had one dominant hand (right = 19, left = 3) based on parents’ report of which hand children used to brush their teeth, cut with scissors, and eat with a spoon, and on our observations of children cutting with scissors and drawing a line.

### Videos of multi-step actions

Participants watched a set of “test” videos to determine their perception of multi-step actions, interspersed with “localizer” videos to determine their baseline neural responses to motor actions (see localizer videos at databrary.org/volume/321/slot/44645/-?asset=231593); all videos were recorded at 30 Hz. Test videos showed three adult actors grasping a hammer and pounding a peg. In half of the videos, actors used an efficient, underhand initial grip that led to an efficient, overhand grip to pound the peg (Fig. 1A). In the other half of the test videos, actors used an inefficient, overhand initial grip that led to an awkward ulnar grip to pound the peg (Fig. 1B). To ensure that other elements of the display could not explain differences between actions, we varied the actor (several actors performed each type of action) and movement trajectories (differed across actors) across displays while keeping constant the visual saliency (identical environments across actions), target (all actors pounded the peg), and outcome (actors were always successful).

All videos began with the actors’ hands placed flat on the table and the hammer handle pointing toward the actors’ non-dominant hand. Actors began moving 2500 ms after the display onset to obtain reliable baseline measures of pupil size and the EEG signal. Each video was 5.4 s long. Videos were edited so that grasping the hammer and pounding the peg occurred at the same time in each clip, regardless of the actor. Moreover, we maintained similar luminance across displays to avoid artifacts in the pupil dilation analysis^42^. Right-handed participants viewed actors pounding the peg with their right hand and left-handed participants viewed actors pounding the peg with their left hand.

Localizer videos were used to map the neural activity involved in action observation by portraying two additional actions to identify features in the neural signal (electrodes, frequencies, and times) that show sensitivity to action observation (see localizer videos at databrary.org/volume/321/slot/44645/-?asset=231603).

Unlike the test videos, the localizer videos do not show grips that are unique to children or adults. In one localizer video, the action starts with an overhand/radial grip and ends with an overhand/radial grip. This action has the same initial and end grips as the inefficient action in the test videos. However, unlike the inefficient action, the action includes a rotatory motion in which the wrist turned out away from the body and the fingers pointed downward toward the body (Figure S1-A). In the other localizer video, the action starts with an overhand/ulnar grip, similar to the efficient action in the test videos. However, unlike the efficient action, it ends with an underhand/ulnar grip (Figure S1B).

### Procedure

As shown in Fig. 1C, participants sat in a child or adult-sized chair in front of a 60-cm widescreen LCD monitor (1920 × 1200 resolution) with the height and orientation of the monitor adjusted to participants’ eye level. We dimmed the room light to improve the quality of the eye-tracking recording. Children were told they are going to watch cartoon movies and movies of actors hammering down a peg. A desk-mounted SMI eye tracker (SensoMotoric Instruments, RED, 120 Hz) recorded gaze location and pupil dilation. We used a 4-point routine to calibrate the tracker, then validated the calibration with a second 4-point routine, and finally used a third 4-point routine at the end of the session to ensure that the calibration did not deviate^50^.

We fitted participants with a child- or adult-sized, wireless, 32-channel EEG cap (Neuroelectrics, ENOBIO 32, 500 Hz). Data were recorded from 32 scalp electrodes at locations of the extended 10–20 system and from two electrodes on the right mastoid. The single-ended voltage was recorded between each electrode site and CMS/DRL electrodes.

Each participant watched 96 videos in total (48 test videos and 48 localizer videos), presented in 8 blocks of 12 videos interspersed with animated cartoons to maintain participants’ motivation and attention. Each block contained the same grasp-to-pound action performed by all three actors. Actors were pseudorandomized within blocks (no more than 3 displays by the same actor consecutively) and the order of blocks was randomized within age groups.

### Eye-tracking: data processing and analyses

*Areas of interest*: We defined 5 rectangular areas of interest (AOIs) for each video frame using SMI BeGaze software (Fig. 2A): the hammer (average size of area within and across videos = 12,397 pixels); the pegboard (30,223 pixels); the actor’s dominant and non-dominant hand (13,618 pixels for right hand; 12,961 pixels for left hand); and the actor’s face (25,112 pixels). The AOIs for the hammer, peg, and dominant hand are essential to capture the action (yellow, red, and orange areas in Fig. 2A)—the hammer is the means to achieve the goal, the peg is the target of the action and the dominant hand is the moving hand. The AOIs for the the actor’s nondominant hand and face are known to attract observers’ gaze^51–53^, but are not essential to capture the action (blue areas in Fig. 2A).

To determine the percentage of time participants’ gaze tracked the action in the videos, we conducted dynamic area-of-interest analyses using the SMI BeGaze software. For each video frame, participants could look at one AOI, two or more AOIs (e.g., when the actor grasped the hammer or pounded the peg), or irrelevant areas (table, wall, etc.). Conflicts between overlapping AOIs were resolved by attributing gaze to the front-most AOI based on depth order. The AOI that most recently moved into the space of another AOI is the front-most. Percent “dwell” time was the total time that the gaze was on an AOI divided by the total tracking time. Percent “fixation” time was the total time that the gaze was on an AOI for at least 100 ms divided by the total fixation time. Frequency of “revisits” was the number of times participants shifted their gaze to an AOI from outside the AOI.

*Scanning patterns*: To test participants’ scanning patterns, we calculated moment-to-moment gaze shifts from one AOI to another. Gaze shifts occurred when participants fixated an AOI and then fixated a different AOI within 100 ms. We counted the number of gaze shifts between each pair of AOIs and averaged them across participants in each age group to obtain a 5 × 5 matrix per age group, where each cell represents the average number of gaze shifts between each pair of AOIs.

*Temporal probability maps*: Looking patterns from movement onset to the initial grasp reflect participants’ attention to the first step in the multi-step action. Thus, for each participant, we determined the probability of looking at the actor’s reach trajectory and grasp of the hammer handle by dividing the number of video frames each pixel was fixated (within a 75-pixel radius of participants’ gaze location to account for detection resolution) by the total number of video frames.

Averaging the probabilities across participants in each age group yielded two “temporal probability maps.” We then compared the maps using an unequal variance *t*-test for each pixel and corrected for multiple comparisons by controlling the False Discovery Rate^54^ with a threshold of *q*(FDR) < 0.05.

*Deep-learning analysis*: We used a deep-learning video classifier^55^ to determine whether participants gathered sufficient visual information to distinguish between the efficient and inefficient action plans. For each participant and trial, we created a “looking video” to represent the visual information gathered in each trial. That is, for each video frame in the looking video, the area within a 75-pixel radius of participants’ gaze was identical to the original display and the rest of the frame was black (Figure S2; all looking videos: databrary.org/volume/321; example: databrary.org/volume/321/slot/25121/-?asset=232029). The resulting looking videos were used as input to the classifier. If participants paid sufficient visual attention to the initial step in the multi-step action, the classifier should be able to correctly distinguish efficient from inefficient actions based on the looking videos.

We applied the classifier separately for each participant. To classify the test videos based on the derived looking videos, we created a deep-learning neural network that combines a pre-trained image classification model, convolutional neural network (CNN), and long-short term memory (LSTM) network. We used the Matlab Deep Learning Toolbox to (1) load a pre-trained GoogleNet model; (2) convert each video to a feature sequence using CNN; (3) create an LSTM network that classifies feature sequences; and (4) assemble a unified classification network using layers from both CNN and LSTM.

For each participant, we randomly chose one trial from a test video with an efficient action plan and one trial from a test video with an inefficient action plan as a “test set.” The remaining 46 trials (23 efficient and 23 inefficient trials) were used as a “training set” for the classifier. We assessed the accuracy of the classifier by comparing its assigned labels to the trials which it was not trained on and the actual labels of the original test videos. Classification accuracy was assessed as the average performance level across 500 randomly chosen trials for the test-set (“leave-one-out” procedure). Significance was assessed by comparing the accuracy levels to a null distribution (*p* < 0.05) obtained by shuffling the labels of the trials (1000 shuffles) and performing the same classification procedure as on the original data.

### Pupil dilation: data processing and analyses

We analyzed pupil dilation as in van Rij, et al.^19^. We used SMI BeGaze software to remove artifacts (blinks and saccades) from the pupil dilation signal. Visual inspection of the data corroborated automatic artifact rejection.

To analyze pupil response in relation to the actor’s movements in the videos, we down-sampled the pupil data to 30 Hz and aligned it to movement onset. “Baseline” pupil size was the average pupil size during the first 1250-ms time window (in the period before movement began, as is customary). For each video frame of each trial, we defined pupil response as a percent of that trial’s baseline pupil size.

### EEG: data processing and analyses

Data were analyzed offline using the EEGLAB tool for MATLAB^56^. Raw EEG data were band-pass filtered offline between 1 and 45 Hz (Butterworth filter, 24 db), and re-referenced offline to the digital average of the two mastoids. The continuous data were segmented into epochs from − 1.5 s to + 2.9 s relative to movement onset in each video. Eye movements and blinks were detected and removed using independent component analysis (ICA^57^). Data were visually inspected for noise (such as movement artifacts) and eye-tracking data were used to exclude times from the EEG data when children did not look at the screen.

*Time–frequency analysis*: Event-related spectral perturbation (ERSP) was computed using a continuous Morlet wavelet transform. For each participant, we computed the logarithm of the power (from 0 to 2.9 s post-movement onset) relative to power during baseline (from − 1.5 to 0 s before movement onset). We defined suppression indices in the various frequencies as the average log-ratio values. A negative log-ratio indicates a suppression in the power of EEG oscillations relative to baseline, whereas positive log ratios indicate enhanced power.

*Action observation localizer*: Participants’ brains differ as do participants’ neural activity while observing actions. Thus, we used an individualized, data-driven approach to determine specific differences in neural activity due to viewing the efficient versus inefficient test video. The EEG signal to the localizer videos provides the frequencies, timing, and channels of neural activity related to action observation for each participant. We focused on significant suppression in the signal during the time between movement onset and hammering in the 6–20 Hz frequency range (frequencies previously reported as associated with action observation in children and adults^3,58^. To this end, a nonparametric cluster analysis was performed on the power in bilateral sensorimotor sites (FC1-5, C1-6, CP1-6) and bilateral occipital sites (O1 and O2) during action observation. Statistical significance was assessed with a well-established procedure that accounts for multiple comparisons^59^. Electrodes with significant suppression were used for classification of data recorded during observation of the test-videos.

*Classification analysis*: We examined whether efficient and inefficient planning could be distinguished based on the neural activity related to action observation for each participant. Thus, for each participant, we constructed a support vector machine (SVM) classifier. The classifier was provided with the label of each trial (efficient or inefficient planning) and the corresponding power suppression in the time and frequency windows showing significant suppression as detected from the localizer data. We used a Matlab implementation of binary SVM^60^ and applied a least-squares cost function (C = 1).

To determine the classification accuracy for each participant, we used a standard leave-one-out classification procedure in which we randomly chose one trial of an efficient and inefficient test video to serve as the “test set” and the classifier was trained on the remaining dataset. Following training, classification performance was assessed by comparing the classifier’s assigned labels to the test set (the trials which it was not trained on) and their real labels. The average performance level across 500 permutations was assigned as the classification accuracy. Similar to the deep learning analysis, significance was assessed by comparing the accuracy levels to a null distribution (*p* < 0.05) obtained by shuffling the labels of the data (1000 shuffles) and performing the same classification procedure as on the original data.

## Supplementary Information

Supplementary Information.
