# Supplementary material for: Children do not distinguish efficient from inefficient actions during observation
Source: Sci Rep. 2021 Sep 13;11:18106. doi: 10.1038/s41598-021-97354-9 (PMC8438080; doi:10.1038/s41598-021-97354-9)
Supplement: Supplementary file 1 — Supplementary Information. [file 41598_2021_97354_MOESM1_ESM.docx]

# Supplemental Figures


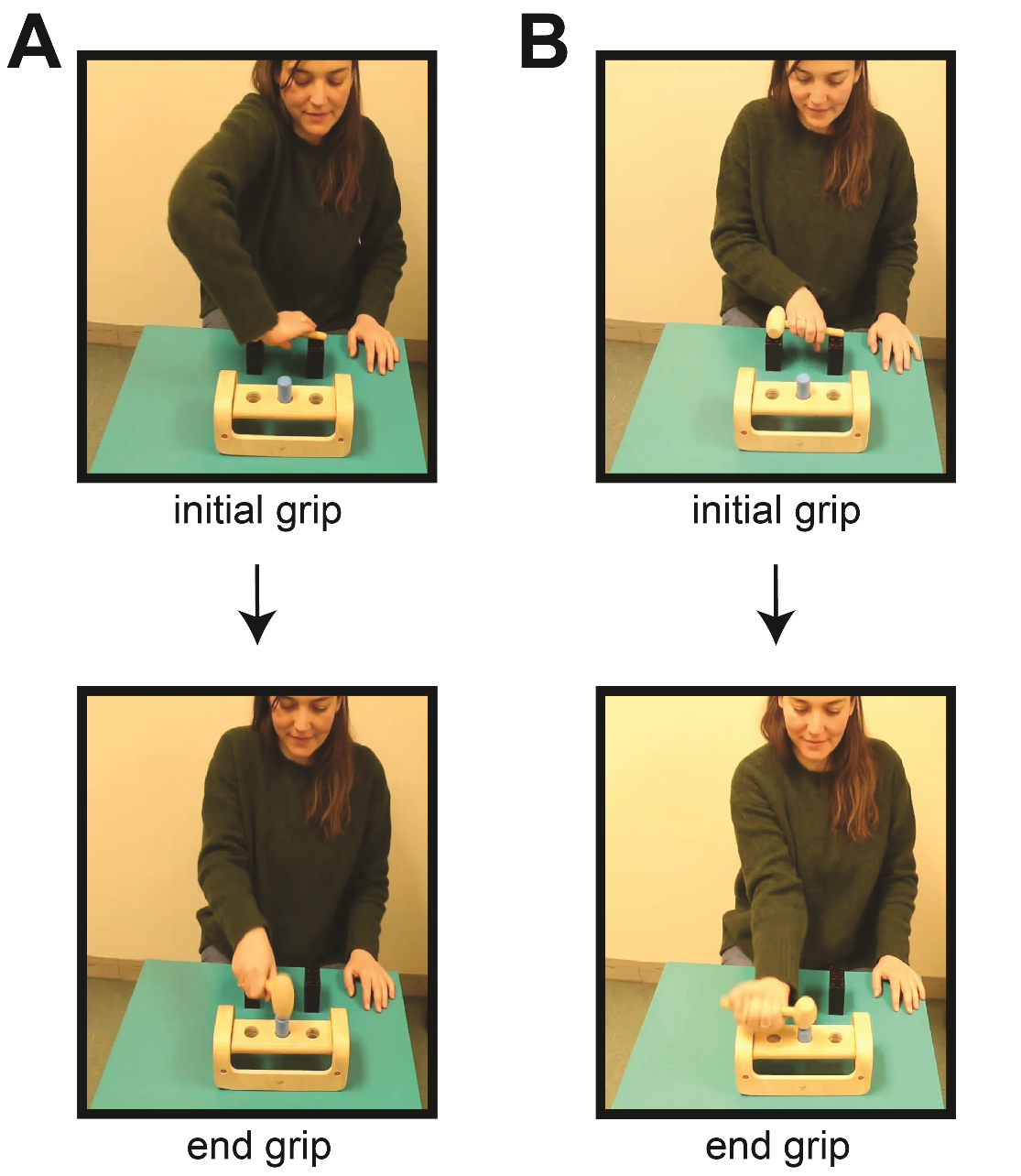


**Figure S1.** Examples of action displays used for EEG localization. **(A)** One action started with an underhand/radial grip and ended with an overhand/radial grip with a rotatory motion in which the wrist turns out away from the body and the fingers point downward and in toward the body. **(B)** Another action started with an initial overhand/ulnar grip and ended with an underhand/ulnar grip.


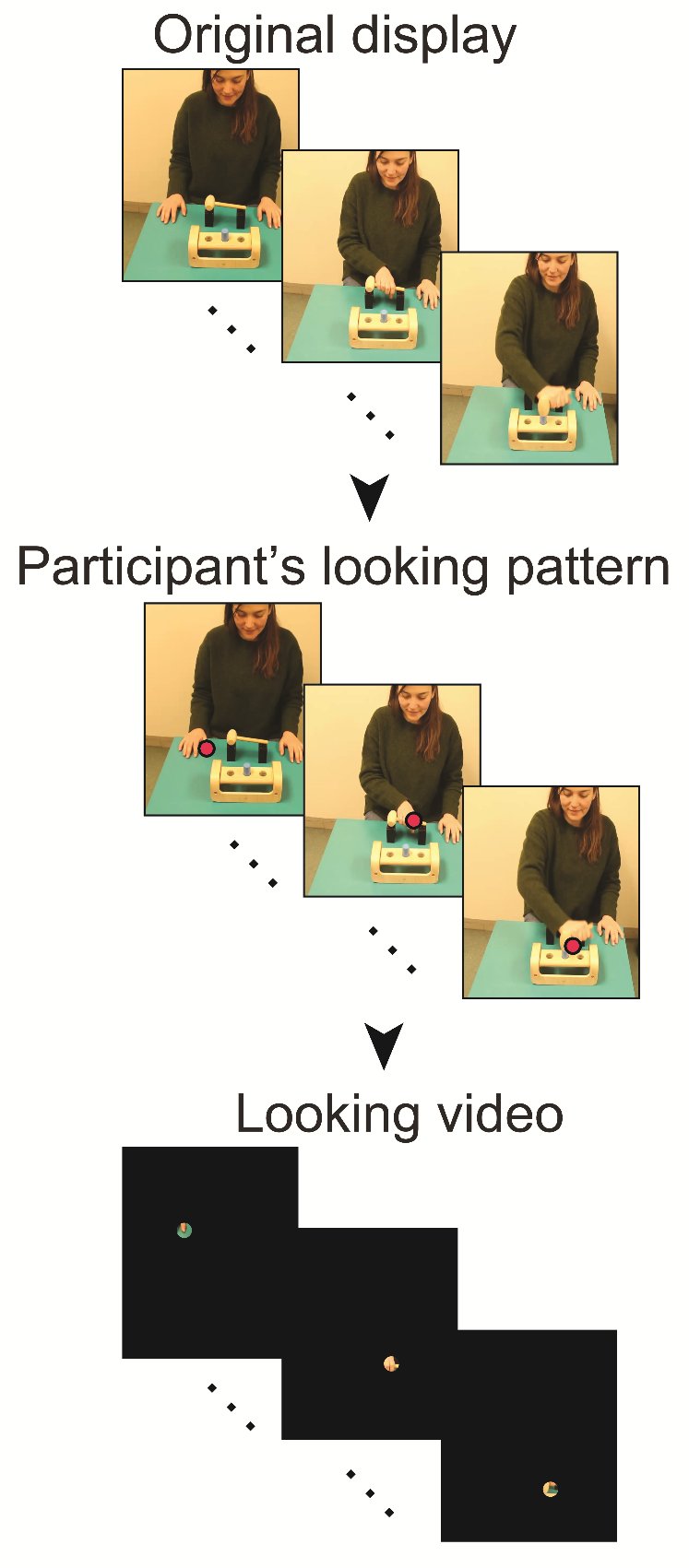


**Figure S2.** Looking videos were created from the original displays based on participants’ looking patterns in each frame. These videos were used as input in the deep learning analysis.
